# *HoxD* expression in the fin-fold compartment of basal gnathostomes and implications for paired appendage evolution

**DOI:** 10.1038/srep22720

**Published:** 2016-03-04

**Authors:** Frank J. Tulenko, Gaius J. Augustus, James L. Massey, Seth E. Sims, Sylvie Mazan, Marcus C. Davis

**Affiliations:** 1 Department of Molecular and Cellular Biology, Kennesaw State University, GA, USA; 2 Cancer Biology Graduate Interdisciplinary Program, University of Arizona, AZ, USA; 3 Department of Ecology and Evolutionary Biology, University of Colorado Boulder, CO, USA; 4 CNRS, Sorbonne Universités, UPMC Univ Paris 06, Observatoire Océanologique, UMR7232 Banyuls, France

## Abstract

The role of Homeobox transcription factors during fin and limb development have been the focus of recent work investigating the evolutionary origin of limb-specific morphologies. Here we characterize the expression of *HoxD* genes, as well as the cluster-associated genes *Evx2* and *LNP*, in the paddlefish *Polyodon spathula*, a basal ray-finned fish. Our results demonstrate a collinear pattern of nesting in early fin buds that includes *HoxD14*, a gene previously thought to be isolated from global *Hox* regulation. We also show that in both *Polyodon* and the catshark *Scyliorhinus canicula* (a representative chondrichthyan) late phase *HoxD* transcripts are present in cells of the fin-fold and co-localize with *And1*, a component of the dermal skeleton. These new data support an ancestral role for *HoxD* genes in patterning the fin-folds of jawed vertebrates, and fuel new hypotheses about the evolution of cluster regulation and the potential downstream differentiation outcomes of distinct *HoxD*-regulated compartments.

One of the challenges of evolutionary morphology is to gain insight into how changes in developmental programs contribute to anatomical diversification in lineages descended from a common ancestor. Hox subfamily transcription factors are critical organizers of regional patterning during embryonic development, and have received much attention for their role in morphological evolution^1^,^2^,^3^,^4^,^5^,^6^,^7^. Invertebrates typically possess a single *Hox* cluster, whereas vertebrates possess at least four *Hox* clusters—designated as *HoxA, B, C,* and *D—*resulting from two rounds of whole genome duplication^8^. Additional duplication events further expanded the repertoire of *Hox* genes in several vertebrate lineages including lamprey (6 clusters: ref. 9), teleosts (8 ancestrally, with patterns of both loss and further duplication within derived clades; refs 10, 11, 12), and paddlefish, a non-teleost actinopterygian^13^. The genes within each *Hox* cluster fall into a series of paralogy groups based on sequence homology. Comparative genomic studies support the hypothesis that the ancestral vertebrate *Hox* cluster included 14 paralogues^8^,^14^,^15^. While there is significant experimental and clinical data describing paralogy groups *1–13*, little is known about the role of *Hox14* paralogues during development due to their loss from the genomes of teleosts and tetrapods thus far sampled^8^,^13^,^15^,^16^.

How paired fins gave rise to limbs during the invasion of land is one of the compelling questions in vertebrate evolution^17^,^18^. This morphological transition involved several key changes in appendage anatomy, including the loss of the dermal skeleton of the fin, and an expansion/remodeling of the distal endoskeleton to form an autopod with digits^19^,^20^ (Fig. 1). *HoxA*/*D* cluster genes are active during both fin and limb development, and over the last two decades, have been the focus of many studies aimed at gaining insight into the evolutionary origin of limb-specific morphologies^21^,^22^,^23^,^24^,^25^,^26^,^27^,^28^,^29^.

In tetrapods, the expression of *HoxD* genes are temporally and spatially dynamic during limb ontogeny, occurring in two phases, an early phase and a late phase, each driven by distinct regulatory elements positioned outside the cluster^30^,^31^,^32^. This has been particularly well-studied in mice, where during the early phase, transcripts of *HoxD1–9* are present throughout the initial limb bud, followed by the sequential, posteriorly-nested activation of *HoxD10–13* during bud outgrowth^33^. This first phase of collinear expression (i.e., *HoxD13* is most posteriorly restricted) patterns the proximal limb (stylopod and zeugopod), and is predominantly regulated by enhancer elements positioned telomeric (3′) to the cluster^33^,^34^. In contrast, during the late phase of expression, *HoxD9–13* are activated in the autopod, forming an inverse collinear arrangement (i.e., *HoxD13* is most broadly expressed) that patterns the digits^33^. The enhancer elements that drive this late phase are interspersed across a region centromeric (5′) to the cluster and have been shown to regulate the expression of *Evx2* and *LNP*, two non-*Hox* genes within the 5′ genomic neighborhood^35^,^36^,^37^. Together, these observations from mice underscore a topological switch between domains of chromatin interaction to pattern the zeugopod and autopod, respectively. Interestingly, one current model for limb development derived from these data posits that this regulatory transition creates a zone of low Hox dosage that manifests anatomically as the small, nodular bones of the wrist and ankle^34^,^38^.

Among non-tetrapod gnathostomes, the distribution of *HoxD* transcripts during paired fin development has been partially characterized in teleosts [zebrafish: refs 21, 24 and 39], a basal actinopterygian [paddlefish: ref. 22], a basal sarcopterygian [lungfish: ref. 40], and two chondrichthyans [catshark and skate: refs 23, 41 and 42]. These *in situ* studies reveal a conserved, collinear pattern of expression in the proximal region of early fin buds, similar to the first phase of *HoxD* expression in tetrapods^18^. In later stages of paired fin development, *HoxD* boundaries shift, suggesting a change in regulatory control. The dynamics of these shifts, however, vary with lineage. In zebrafish, a late phase of *HoxD11a–13a* expression appears to extend into the mesenchyme of the dermal fin-fold, creating a patterning compartment in which the anterior and posterior boundaries of each paralogue are in register^24^. In contrast, current data sets for paddlefish and catshark indicate that 5′ *HoxD* transcripts form an inverse collinear profile^22^,^23^,^41^. In paddlefish, this late phase overlaps early expression at the site of distal radial formation in the mid-fin, and has been interpreted as evidence of a deep developmental homology (*sensu* refs 43 and 44) between the distal radials and digits^22^ (but see ref. 38). In catshark, late phase *HoxD* transcripts localize to the distal margin of the fin^23^,^41^, and unlike actinopterygians are spatially separated from the early phase of expression^45^. Overall, these data from non-tetrapods suggest an evolutionary scenario in which distinct proximal and distal *HoxD* patterning compartments were already in place in the paired fins of ancestral gnathostomes^18^,^23^,^45^.

Recent studies have focused on testing whether the regulatory landscapes that control *Hox* expression in fins and limbs are functionally conserved. Significantly, this work has shown that several of the enhancer elements that drive digit expression in mice are represented in non-tetrapods^17^,^25^,^29^,^35^,^37^,^46^, and that a subset of these homology regions are not only active distally during paired fin development [zebrafish CsB: ref. 25; zebrafish Island I: ref. 29; Gar CsB and Island I: ref. 29], but when inserted into mice drive reporter expression in the proximal autopod [skate CsB: ref. 25; zebrafish CsB: ref. 25; pufferfish BAC clones: ref. 28] and digits [Gar CsB and Island I: ref. 29]. Additionally, there is evidence that trans-acting factors are at least partially conserved between fins and limbs, as a tetrapod specific element (mouse CsC) has been shown to drive distal fin expression when inserted into zebrafish^26^. Together, these results provide evidence that the origin of limb-specific morphologies involved modification of an ancient, conserved regulatory architecture for *HoxD* activation already in place in fins.

Current models for the fin to limb transition that integrate these data focus on the role of *HoxD* genes in patterning the endoskeleton, though with alternative perspectives on the relationship between cluster regulation and anatomical homology^17^,^18^,^28^,^29^,^38^,^45^. In these models, the fin-fold is considered a Hox-free zone, and its formation is thought to interrupt the ectodermal/mesenchymal signaling interactions that determine the relative size of a Hox-patterned endoskeletal mesenchyme^17^,^23^,^24^. Key to further informing models of appendage evolution is a better understanding of the phylogenetic distribution of *HoxD* expression relative to the proximo-distal fin compartments in basal gnathostomes. Herein, we characterize the expression of *HoxD* genes, as well as the cluster-related genes *Evx2* and *LNP*, in a basal actinopterygian, the American paddlefish *Polyodon spathula*. Our results demonstrate a posteriorly restricted, collinear pattern of nesting in the early fin buds that includes *HoxD14Beta*, a gene previously thought to be insulated from canonical *Hox* regulation in the mesoderm^14^,^47^. Additionally, we show that in later stages of fin development, the collinear expression profile established in early fin buds is maintained along the site of distal radial formation, with no “autopodial-like” anterior expansion of *HoxD13* (contra a previous report for *Polyodon*^22^), *Evx2,* or *LNP*. Most significantly, we observed a proximo-distal dynamic to *HoxD* expression in which late phase transcripts of both 3′ and 5′ paralogues extended into the fin-fold mesenchyme, co-localizing with *And1*, the elastoidin component of the actinotrichia^48^. In order to determine the phylogenetic distribution of this condition, we compared the expression domains of *And1* and *HoxD12* in a representative chondrichthyan, the lesser-spotted catshark *Scyliorhinus canicula*. These *in situs* revealed an overlap between distal *HoxD* and *And1* expression, much like that of paddlefish, suggesting HoxD-positive cells contribute to fin-fold specific tissues in both taxa (the ceratotrichia and actinotrichia of chondrichthyans and actinopterygians, respectively). Our results support an ancestral role for *HoxD* genes in patterning the fin-fold, bringing new information to current models for fin/limb evolution and fueling novel hypotheses about the relationship between cluster regulation and appendage morphology.

## Results

### Expression of *HoxD* cluster genes in paddlefish paired fins

A whole genome duplication event occurred in the paddlefish lineage approximately 42 million years ago, resulting in the formation of unique *Alpha* and *Beta HoxD* clusters^13^ (Fig. 2a). In order to determine if both clusters are transcriptionally active during development, we surveyed our *Polyodon* transcriptome assembly using published genomic BAC sequences for specific *HoxD Alpha* and *Beta* paralogues^13^. These blast searches identified both *Alpha* and *Beta* sequences of *HoxD8*, *HoxD12,* and *HoxD13,* and *Beta* sequence of *HoxD11.* Unexpectedly, we also identified transcripts of *HoxD14*, a *Beta* cluster gene previously hypothesized to be inactive during appendage patterning in vertebrates^14^,^47^.

In order to characterize the spatiotemporal expression dynamics of *HoxD* cluster genes during paired fin development, we performed a series of *in situ* hybridizations using probes targeted against either *Alpha* or *Beta* paralogues. Because the distribution of labeling appeared similar between genes from duplicated clusters (compare Fig. 2b and Supplemental Fig. 1), we limit the descriptions presented here to the *HoxD Beta* cluster only. It is worth noting, however, that we cannot rule out the possibility of probe cross reactivity due to high sequence similarity between a given set of paralogues (e.g., *HoxD11Alpha* and *HoxD11Beta*), and that positive staining may reflect their combined distribution of transcripts. Additionally, we could not assign an *Alpha/Beta* identity to the *HoxD4* clone used in our analyses in the absence of available genomic sequence from the 3′ end of each duplicated cluster^13^.

In Stage 42 embryos, transcripts of *HoxD4* and *HoxD8Beta* were detected along much of the length of the pectoral fin buds (Fig. 2b). In contrast, transcripts of *HoxD11Beta*–*14Beta* were more restricted in distribution, forming a collinear, posteriorly nested pattern of expression that mirrored the 3′–5′ arrangement of paralogues within the cluster (Fig. 2a,b). In more advanced embryos (Stages 44 and 46), the expression domains of *HoxD4*, *HoxD8Beta*, and *HoxD11Beta*–*HoxD13Beta* persisted in the middle of the fin following outgrowth of the distal fin and fin-fold (see arrowheads in Fig. 2b). Although an anterior expansion of *HoxD13* expression in stage 46 fins has been reported in *Polyodon* (ref. 22 and see additional discussion in Supplemental Fig. 1), we observed that the collinear pattern of *HoxD* nesting established in early fin buds was maintained through later stages (Fig. 2b, Supplemental Fig. 1, and Supplemental Fig. 2). We also examined the distribution of *LNP* and *Evx2* to infer if these non-*Hox* genes are coordinately regulated with 5′*HoxD* paralogues, as in mice where *Evx2* and *LNP* co-localize with *HoxD13*^35^. In paddlefish, *Evx2* expression was restricted to the posterior most region of the fin and appeared similar in distribution to *HoxD14Beta* (Fig. 2b). *LNP*, in contrast, was detected throughout the pectoral fins, with no distinct regionalization (Fig. 2b). Pelvic fins mirrored the 5′ *HoxD* and *Evx2* expression patterns observed in pectoral fins (Fig. 2c). Transcripts of the more 3′-ward paralogues *HoxD4* and *HoxD8Beta*, however, were difficult to detect in whole mount.

### *HoxD* expression in the fin-fold compartment of paddlefish

**Figure 1:**
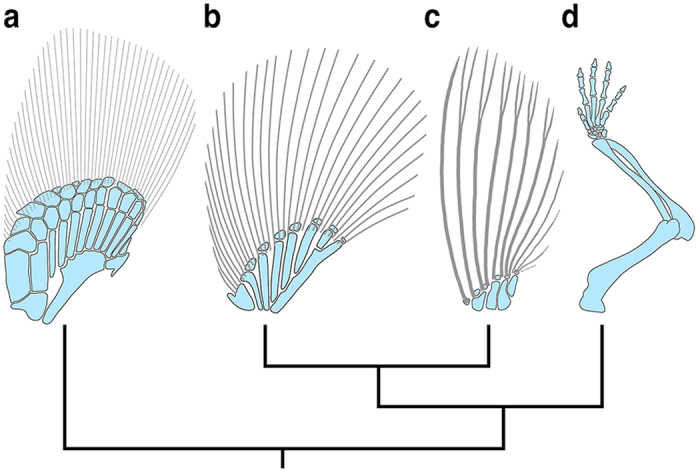
Pectoral appendage skeletons for representative vertebrates. (**a**) Chondrichthyan (catshark - *Scyliorhinus canicula*). (**b**) Non-teleost actinopterygian (paddlefish - *Polyodon spathula*). (**c**) Teleost (zebrafish - *Danio rerio*). (**d**) Tetrapod (mouse – *Mus musculus*). Endochondral skeleton in blue, dermal skeleton in gray. Anterior is left, distal is up for all figures. Figures not to scale.

During limb development, *HoxD* genes are expressed in well-characterized proximal and distal zones that correspond to the stylopod/zeugopod and autopod, respectively. Given that fins are highly regionalized along the proximo-distal axis into endoskeletal radials (proximally) and a fin-fold/dermal skeleton (distally) (Fig. 1), we sought to characterize the distribution of *HoxD* transcripts relative to the early patterning zones that give rise to these morphologically distinct compartments. *Actinodin* genes encode non-collagenous (elastoidin) components of the actinotrichia and provide an early molecular marker for cells contributing to the fin-fold^48^. We identified a homologue of *Actinodin1 (And1)* in paddlefish, which we used for both single and double colorimetric *in situs* along with the pre-chondrogenic marker *Sox9* to visualize the early endoskeletal radials (Fig. 3). In Stage 41 embryos, *And1* transcripts were detected along the apical margin of the pectoral fin buds, forming a labeling boundary just distal to the *Sox9* expression domain (Fig. 3a). In later stage fins (Stage 42–45), the relative position of the *And1*/*Sox9* boundary was maintained despite considerable growth in both compartments. Cross sections through developing fins at these stages revealed *And1* transcripts in both the fin-fold mesenchyme and the ectoderm adjacent to the basement membrane (Fig. 3b). Notably, at stage 45 the proximal margin of *And1* labeling extended beyond the distal, lateral margins of *Sox9* labeling, presaging the ultimate arrangement of the dermal fin supports and cartilaginous fin radials described for mature fins (see Fig. 3 in refs 49 and 50).

**Figure 2:**
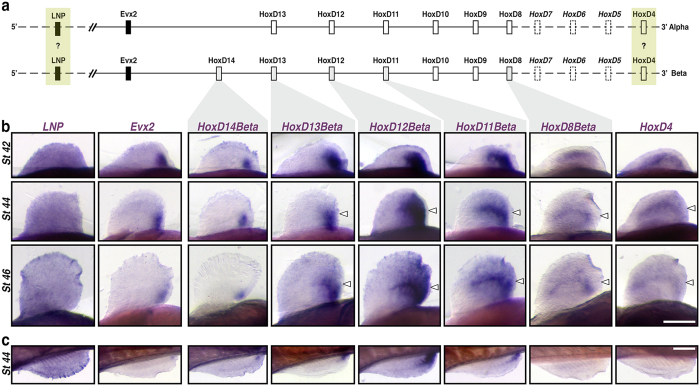
Expression of HoxD cluster genes in paddlefish paired fins. (**a**) Schematic representation of Alpha and Beta HoxD clusters based on ref. 13. Gene key: Open boxes – Hox genes; closed boxes – non-Hox genes; solid lines – genes characterized and attributable to either Alpha and Beta clusters based on published BAC clones; yellow boxes – genes cloned but not attributable to a specific cluster; Dashed boxes – uncharacterized genes. (**b**) Pectoral fin whole-mount *in situ* hybridizations for *LNP*, *Evx2*, *HoxD14Beta*, *HoxD13Beta*, *HoxD12Beta*, *HoxD11Beta*, *HoxD8Beta*, and *HoxD4* from stages 42 (early fin bud), 44 (onset of endoskeletal radial differentiation), and 46 (differentiated fin – onset of feeding larva)^49^. Pectoral fins in ventral view, anterior to the left, distal is up; Genes are shown in columns, and developmental stages in rows. Open arrowheads denote the position of distal radial formation along the A-P axis, where *HoxD* expression persists^22^ following outgrowth of the fin-fold. (**c**) Pelvic fin whole-mount *in situ* hybridizations comparable to (**b**) for stage 44. Pelvic fins in medial view, anterior to the left, distal is down. Scale bars = 200 nm.

**Figure 3:**
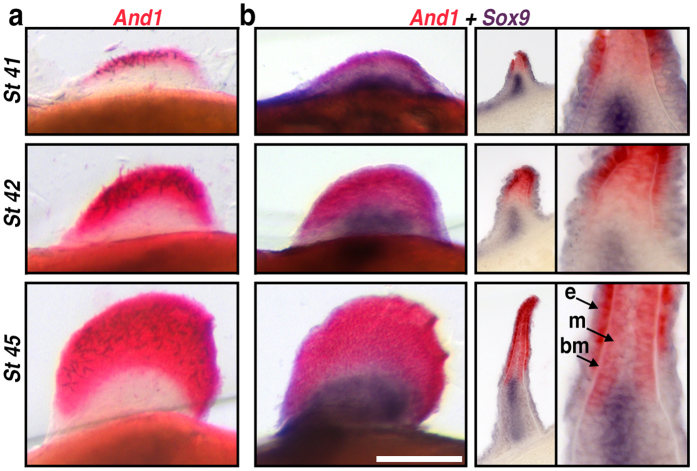
*Actinodin1* expression in the fin-fold compartment of paddlefish. (**a**,**b**) Pectoral fin *in situ* hybridizations in whole-mount, representative cross sections, and magnifications. Stages are shown in rows **(a)** *Actinodin1 (And1)*, an early molecular marker for cells contributing to the fin-fold. *And1* transcripts (red) appear in the presumptive distal fin and fin-fold of early fin buds (stage 41) and persist as the fin-fold elongates (stages 42 and 45). (**b**) Double *in situs* for the pre-chondrogenic marker *Sox9* (purple) and *And1* (red) reveal that endochondral and dermal compartments remain separate throughout fin development. Cross sections (and magnification) reveal *And1* transcripts in both the fin-fold mesenchyme (m) and the ectoderm (e) adjacent to the basement membrane (bm). The slight proximo-distal overlap between *And1* and *Sox9* expression presages the arrangement of the dermal fin skeleton later in development. Scale bars = 200 nm.

**Figure 4:**
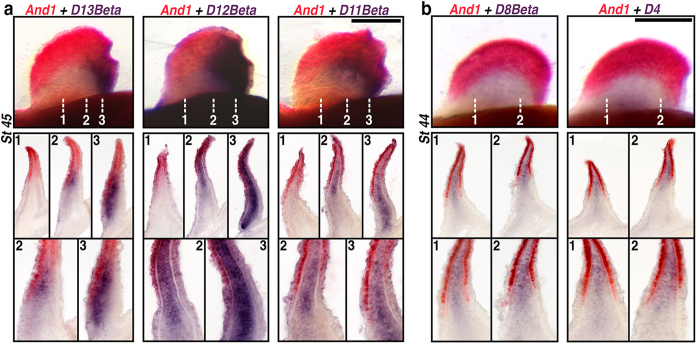
*HoxD* expression in the fin-fold compartment of paddlefish. (**a**,**b**) Pectoral fin double *in situs* for HoxD genes (purple) and *And1* (red) in whole-mount (top row), representative cross sections (middle row) and magnifications of select sections (bottom row). Numbers mark planes of section. Genes are shown in columns. (**a**) Co-expression of 5′ Hox genes *HoxD13Beta*, *HoxD12Beta,* and *HoxD11Beta* (purple) with *And1* (red) reveal the posteriorly-restricted pattern observed in 5′ HoxD single *in situs* (compare with Fig. 2b). Significantly, cross sections confirm that 5′ HoxD expression extends distally to overlap the *And1* expression domain. 5′ HoxD transcripts were not detected in the anterior fin (sections labeled 1). More posterior sections reveal expression throughout the fin-fold mesenchyme, with a proximal boundary that roughly aligned with ectodermal *And1* (sections labeled 2). In sections near the caudal margin of the fin, 5′ HoxD expression is continuous between the fin-fold and proximal fin mesenchyme (sections labeled 3). (**b**) Co-expression of the more 3′-ward HoxD genes *HoxD8Beta*, and *HoxD4* (purple) with *And1* (red) reveals transcripts of both Hox paralogues within the fin-fold mesenchyme and aligned proximally with the *And1* labeling boundary in the ectoderm (sections labeled 1, 2). Anterior is left, distal is up for all whole mounts. Ventral is left, distal is up for all sections. Scale bars = 200 nm.

Our *And1*/*Sox9* data make visible the proximal boundary of the fin-fold patterning compartment at various stages of pectoral fin development, and show that prechondrogenic cells are excluded from the population of mesenchyme distal to this boundary. We next performed a series of double colorimetric *in situs* to determine if *HoxD* paralogues co-localize with *And1* in the fin-fold mesenchyme. In whole mount pectoral fins at Stage 45, transcripts of *HoxD11Beta*–*HoxD13Beta* formed the same posteriorly-restricted pattern of nesting along the mid-fin (Fig. 4a) as in single *in situs* (Fig. 2b). Significantly, the expression of these 5′ *HoxD* paralogues extended distally to overlap with the *And1* expression domain. Cross sections revealed differences in the profile of *HoxD* and *And1* boundaries, depending on their position along the anterior-posterior axis (Fig. 4c). Whereas no 5′ *HoxD* transcripts were detected in the anterior portion of the fin, in more posterior sections *HoxD11Beta*–*HoxD13Beta* were expressed throughout the fin-fold mesenchyme, with a proximal boundary for strongest labeling that roughly aligned with that of *And1* in the ectoderm. In sections near the caudal margin of the fin, no such alignment was observed, as *HoxD11Beta*–*Hox13Beta* labeling was continuous between the fin-fold and proximal fin mesenchyme.

In mice, early phase HoxD patterning of the proximal limb is primarily driven by the 3′ telomeric landscape and involves the upregulation of paralogues along the length of the cluster (i.e., *HoxD1–13*)^33^,^34^. Regulatory control over *HoxD* expression then transitions to the 5′ centromeric landscape to pattern the autopod, where only *HoxD9–13* (i.e., those paralogues neighboring the 5′ end of the cluster) are active^33^,^37^. In paddlefish, our *in situ* data demonstrate that 5′ *HoxD* paralogues are expressed in the fin-fold (Fig. 4a), which like the autopod in mice represents the distal-most compartment of the appendage. In order to determine if *HoxD* expression in the fin-fold is restricted to genes at the 5′ end of the cluster (similar to late phase autopod patterning), we compared the expression of *And1* and the more 3′-ward *HoxD* paralogues *HoxD4* and *HoxD8Beta*. In whole mount fins, strong *And1* labeling in the ectoderm made it difficult to discern 3′ *HoxD* labeling within the fin-fold. In section, however, transcripts of both *HoxD4* and *HoxD8Beta* were detected in the fin-fold mesenchyme (Fig. 4b), and like the 5′ paralogues examined (Fig. 4a), aligned proximally with the *And1* labeling boundary in the ectoderm.

### *HoxD* expression in the fin-fold compartment of chondrichthyans

**Figure 5:**
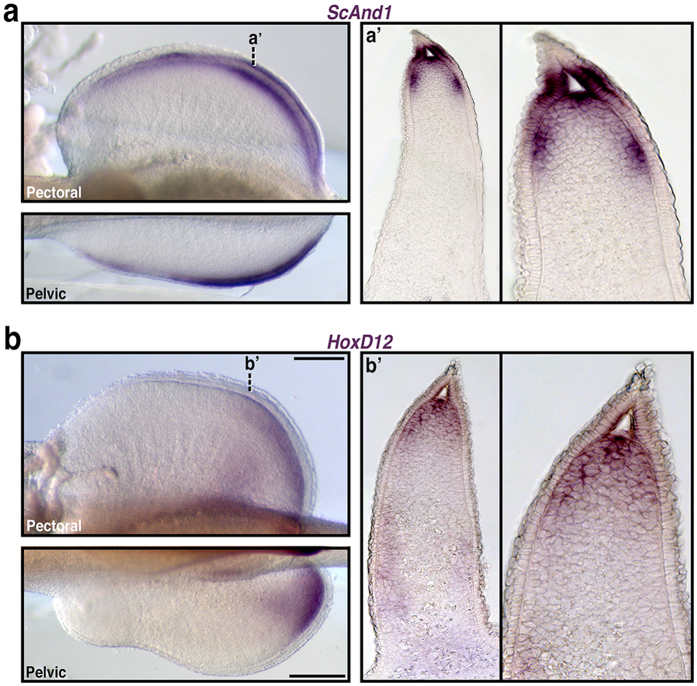
HoxD expression in the fin-fold compartment of catshark. (**a**,**b**) *In situ* hybridizations in whole-mount and representative cross sections. (**a**) *In situ* expression for the catshark *Scyliorhinus canicula Actinodin1* homologue (*ScAnd1*), shown to be an early molecular marker for cells contributing to the fin-fold in teleosts. Whole-mount *ScAnd1* expression in Stage 30 pectoral and pelvic fins is restricted to the distal fin. (**a**’) Cross section of *ScAnd1* Stage 30 pectoral fins (and magnification) reveal *ScAnd1* positive cells mark the boundaries of the fin-fold with both mesenchymal and ectodermal expression domains. (**b**) *In situ* gene expression for *S. canicula HoxD12.* Whole-mount *HoxD12* expression in Stage 30 pectoral and pelvic fins form distinct proximal and distal domains. (**b**’) Cross section of *HoxD12* Stage 30 pectoral fins (and magnification) reveal overlap with the distal domain of *ScAnd1* (compare to **a**’), indicating *HoxD* positive cells in the fin-fold. For all whole mounts: anterior is left, distal is up in pectoral fins, distal is down in pelvic fins. Dashed lines correlate to plane of section in (**a**’,**b**’). Scale bars = 200 nm.

To test whether *Hox* patterning of the fin-fold compartment is a derived condition of paddlefish or a more general feature of gnathostomes, we extended our analysis to the lesser-spotted catshark *Scyliorhinus canicula* as a representative chondrichthyan. Chondrichthyan dermal fin skeletons are composed of ceratotrichia, which are histologically and chemically similar to actinotrichia^51^. Blast searches of the catshark transcriptome assembly returned a putative *Actinodin* homologue, which we identified as *ScAnd1*. Whole mount *in situs* revealed *ScAnd1* expression in the distal pectoral, pelvic, and median fins (Fig. 5a and Supplemental Fig. 3), consistent with the distribution of labeling in actinopterygians (this study, and ref. 48). Cross sections through Stage 30 pectoral fins confirmed that *ScAnd1* expression was both mesenchymal and ectodermal and marked the boundaries of the fin-fold (Fig. 5a).

In catshark, 5′ *HoxD* genes are expressed in early and late waves, patterning the proximal and distal regions of the fin, respectively^23^. In order to determine if the distribution of late phase *HoxD* transcripts extends into the fin-fold compartment, we compared the expression of *HoxD12* with our *ScAnd1* results. In Stage 30 pectoral and pelvic fins, *HoxD12* labeling formed distinct domains proximally and distally, consistent with previously published results^23^ (Fig. 5b). Significantly, cross sections showed considerable overlap between the distal domain of *HoxD12* and *ScAnd1*, indicating *HoxD* expression in the fin-fold mesenchyme of catshark (Fig. 5b).

## Discussion

In this study, we have characterized the expression of *HoxD* and *HoxD* cluster-related genes during paired fin development in the American Paddlefish *Polyodon spathula.* These results demonstrate an early collinear arrangement of 5′ *HoxD* nesting, similar to that of other vertebrates^21^,^22^,^23^,^24^,^33^ and consistent with a model in which the first phase of *HoxD* activation is part of a conserved pathway for establishing *Shh* in the ZPA and appendage asymmetry^52^,^53^,^54^,^55^. Our results also demonstrate that in more advanced fins, collinear *HoxD* nesting is maintained along the presumptive site of distal radial formation following outgrowth of the fin-fold. This persistent mid-fin expression resembles the later dynamics of early phase patterning in limbs, where *HoxD* expression in the nascent limb bud continues in the fore-limb forming territory following outgrowth of the autopod^33^,^38^. Notably, we did not observe an extended anterior sweep of expression of *HoxD13* (*contra* a previous report^22^), *HoxD14*, or the cluster-related genes *Evx2* or *LNP,* in any of the stages examined. These observations provide evidence that the inverse collinear profile considered a hallmark of digit patterning in tetrapods may not be present in paddlefish (or a more inclusive clade, see ref. 24).

In addition to early patterning of the proximal fin bud, our paddlefish *in situ* results reveal a late phase of *HoxD* expression that co-localizes with *And1* in the mesenchyme of the fin-fold, a region absent of pre-chondrocytes. These new data, together with a report of late *HoxD* expression in the fin-fold of zebrafish^24^, suggest this character may be shared among actinopterygians. Chondrichthyans provide a critical outgroup for testing this hypothesis. Our data for catshark confirm the spatially distinct early and late phases of *HoxD* activation described by Freitas and colleagues^23^, and demonstrate overlap between the late distal phase of *HoxD12* labeling and that of *And1*. In tetrapods, most studies of Hox function during limb development have focused on endoskeletal patterning and the effects of gene abrogation on the formation of the stylopod, zeugopod, and autopod^56^,^57^,^58^,^59^,^60^,^61^,^62^. Recent work, however, has shown that *Hox* genes have a broader role in limbs, and are required for normal muscle and tendon formation independent of skeletal phenotype^62^. While the anatomical outcome of *HoxA/D* loss in non-tetrapods remains elusive, the co-localization of *HoxD* and *And1* labeling in a representative actinopterygian and chondrichthyan provide evidence of Hox-positive cells contributing to fin-fold specific tissues (i.e., the elastoidin component of the actinotrichia in paddlefish and ceratotrichia in catshark), and suggest an ancient role for *Hox* genes in integrating aspects of appendage formation beyond endoskeletal patterning.

Our unexpected find, the first report of appendicular expression of *HoxD14Beta*, is *contra* recent hypotheses that *Hox14* paralogues are isolated from canonical regulation of the cluster^14^,^47^. Instead, our results demonstrate that transcripts of *HoxD14Beta* are posteriorly-restricted within the fins, and together with other cluster paralogues form a collinear arrangement of nesting, as would be expected with canonical early phase regulation. These results suggest an ancestral role for *HoxD14* in appendage patterning in gnathostomes, one that has been lost in most lineages^14^,^47^ yet retained, at least in terms of expression, in paddlefish.

Current evidence from comparative gene expression, genomics, and transgenic assays support a model in which bimodal regulation of the *HoxD* cluster is partially conserved between fins and limbs^25^,^26^,^28^,^29^,^46^. The apparent antiquity of this regulatory strategy^28^, together with the data presented here, raise the intriguing and underexplored possibility that *HoxD* patterning of the proximal fin and fin-fold may reflect separate controls from the 3′ and 5′ landscapes, respectively. In tetrapods, late phase *HoxD*-related expression in the autopod is limited to *HoxD9–13* (those paralogues nearest the 5′ end of the cluster) and the 5′ flanking genes *Evx2* and *LNP,* both of which are co-regulated with *HoxD13*^33^,^35^. We predicted a similar restriction in cluster activity in the fin-folds of paddlefish, but found transcripts of the 3′-ward members *HoxD4* and *HoxD8Beta* throughout the fin-fold mesenchyme. This difference, combined with a lack of anteriorized *HoxD13, Evx2* and *LNP,* may reflect lineage specific variation in landscape/cluster interactions between tetrapods and paddlefish, or more generally actinopterygians^24^. Additional comparative data characterizing the expression of 3′ *HoxD* paralogues during fin development in a chondrichthyan will inform inferences about ancestral patterns of cluster activation, and fuel new hypotheses as to the regulatory dynamics of the 3′ and 5′ landscapes during appendage formation in non-tetrapods. Interestingly, current models for limb development posit that chromatin re-organization between early (proximal) and late (distal) *HoxD* expression creates an intermediate zone of low Hox dosage that manifests anatomically as the small bones of the wrist^32^,^34^,^38^. In fins, a series of small, endochondral bones (distal radials) often separate the proximal radials and fin-fold (e.g., Fig. 1). An ancestral role for *HoxD* genes in patterning the distal fin compartment makes it tempting to speculate that a similar mechanism may explain appendage morphologies in non-tetrapods. However, not all patterns meet the predictions of this model (e.g., strong *HoxD* labeling in the presumptive site of distal radial formation in paddlefish), suggesting that modulation of Hox dosage and its effects on cell behavior in the context of local signaling environments differ across lineages. Our results demonstrate that further comparative work, with an increasing focus on basal gnathostomes, will be necessary to elucidate the ancestral roles of Hox genes in appendage patterning, roles that may involve a great flexibility in skeletogenic differentiation outcomes.

## Methods

### Animal husbandry and staging

Embryos of the American Paddlefish *Polyodon spathula* were obtained from Osage Catfisheries Inc. (Osage Beach, MO, USA), and were raised at 18 °C in recirculating large-volume freshwater tanks (pH 7.5 ± 0.2, salinity of 1.0 ± 0.2ppt), fixed overnight in Carnoy’s solution, and dehydrated to 100% ethanol for storage at −20 °C. Embryos of the catshark *Scyliorhinus canicula* were produced at the Biological Model facility of the Roscoff Marine Station, and raised at 17 °C in oxygenated sea water, fixed for 16 hours in 4% PFA, and dehydrated to 100% Methanol prior to storage at −20 °C. Animals were staged according to refs 49, 63, 64, 65.

All experiments and animal care in this study were performed in accordance with the approved institutional guidelines and regulations of the Institutional Animal Care and Use Committee (IACUC) of Kennesaw State University (approved protocol #12-001).

### Transcriptome

Paddlefish larvae from early pectoral fin development stages (stages 41–44)^63^ were preserved in RNAlater and then dissected into pectoral fin and reference tissue (operculum) samples. After RNA library construction, Illumina^TM^ sequencing of the transcriptome was performed by the Centre for Applied Genomics – Toronto (Toronto, Canada) producing a total of 65,682,405 pectoral fin raw reads (72 bp nonpaired). We used the Trinity program (default settings)^66^ to assemble 92,446 likely transcripts. Transcripts were annotated and validated against the National Centre for Biotechnology Information (NCBI) non-redundant (nr) database using BLAST (cut off E value of 10^−5^).

### cDNA synthesis for cloning

RNA was isolated from Stage 26–46 paddlefish embryos with Trizol reagent (Invitrogen) plus the PureLink^TM^ Micro-to-Midi Total RNA Purification System (Invitrogen), and used as template to generate single-strand cDNA with the Superscript III First Strand Synthesis kit (Invitrogen), all per manufacturer’s instructions. Paddlefish primers were designed from transcriptome sequences as follows (F, forward; R, reverse): *Evx2:* CCGGAAGTCTTCCATACCCTTT (F), CCCTTCACACAACCTAACTGACG (R); *Sox9:* CTCGATCCCTTCATGAAGATGAC (F), GATGTGAGTTTGCTCAGATCCG (R); *LNP:* TCCTGAGGAATGGACTGCAA (F), GAGTCTTCCGAGCAGGATTTAGAA (R); *HoxD4:* TGGATCCTAAATTTCCACCTTGCG (F), GCACAGTTTGTAAATGCTGGCTG (R); *HoxD8Beta:* ACTACGACTGTCAGTTTCCACGG (F), CGTCCTTTGGTAGTGAAGATGGGAAT (R); *HoxD12Alpha:* TTTGAATTTCCCTAACCCGGACTC (F), GACGGCATTGTCATGTTTAAGTTG (R); *HoxD12Beta:* TTTGAATTACTCTTCCCCGGACTC (F), CATGGCTGGTTTGAGTCGACAG (R); *HoxD14Beta:* TATGGATGGATCCCAGTTCTCC (F), CGTCTCACCTGTCTTTCTGTCA (R); *And1:* CTCGTACCCTGCGTAGTTACTG (F); CTCACCAAGCCGCTGTAACC (R). Primers for catshark were as follows: *HoxD12:* TATTTCGCCAACCTGCGTCC (F), CTTGTTGGTCGCTCAGGTTCAG (R); *ScAnd1:* TGGCAGAGCAGAACCATGTG (F), GTCAGGATCTTGAACACCTTGGTC (R).

PCR products were cloned into pGEM®-T Easy Vector (Promega), which was purified and sequenced using Genscript DNA Sequencing Services. All sequences were analyzed using Unipro UGENE and BLAST (http://blast.ncbi.nlm.nih.gov/Blast.cgi). Orthology for the *Actinodin* family members was confirmed using maximum likelihood in Mega6.06. GenBank accession numbers for this work are KU744647-KU744655. For *in situ* hybridization experiments, the entire PCR product obtained from each primer set was used as template for *in vitro* transcription (see below). Plasmids containing paddlefish fragments of *HoxD11Alpha, HoxD11Beta, HoxD13Alpha,* and *HoxD13Beta* were obtained through Life Technologies Gene Art Services and were based on published genomic BAC clones^13^. All new probes generated during this study ranged from approximately 480 to 900 base pairs in length. Paddlefish probes for *HoxD4*, *HoxD8Beta*, and *HoxD14Beta* were complementary to regions of coding exons one and two, including the homeodomain. Probes for *HoxD11Alpha* and *HoxD11Beta* were complementary to a region of coding exon one, coding exon two, including the homeodomain, and 3′UTR. Probes for *HoxD12Alpha*, *HoxD12Beta*, *HoxD13Alpha*, and *HoxD13Beta* were complementary to the first coding exon and excluded the homeodomain. Probes for *Sox9*, *And1*, and *LNP* were targeted against coding region. Probe for *Evx2* was targeted against coding region and 3′ UTR. Probes *HoxD13-EF527821* and *ScHoxD12* were from Refs 22, and 23 respectively.

### *In situ* hybridization

Plasmids were linearized for probe template using restriction enzymes (New England Biolabs, NEB) and any resultant 3′ overhangs were blunted using DNA Polymerase I, Large Klenow (NEB). Probe synthesis was carried out using SP6, T7, or T3 RNA polymerases (Promega) and either Digoxigenin or Fluorescein RNA labeling mixes (Roche) per manufacturer′s instructions. Whole-mount *in situ* hybridizations were as described in ref. 67 at a hybridization temperature of 69–70 °C, with the following modifications for double colorimetric *in situs*: Both Digoxigenin (*Sox9, HoxD4, HoxD8Beta, HoxD11Beta–HoxD13Beta*) and Fluorescein (*And1*) labeled probes were included during the hybridization step. Digoxigenin-labeled probes were detected with Anti-Digoxigenin-AP, Fab fragments (1:2000 dilution; Roche) and developed with BM-Purple (Roche). Following the first color reaction, embryos were incubated in 0.1M Glycine (pH 2.2) for 30 minutes to inactivate AP, and then washed thoroughly with MABT. Fluorescein-labeled probes were detected with Anti-Fluorescein-AP, Fab fragments (1:1000 dilution; Roche) and developed using Fast Red Tablets (Roche) per manufacturer’s instructions.

For paddlefish, the total number of antisense *in situ* specimens examined were as follows: *HoxD4:* St41–43 (n = 5), St44–45 (n = 8); St46–1dps (n = 4); *HoxD8:* St41–43 (n = 9), St44–45 (n = 4); St46–1dps (n = 3); *HoxD11Alpha:* St41–43 (n = 4), St44–45 (n = 3); St46–1dps (n = 4); *HoxD11Beta:* St41–43 (n = 11), St44–45 (n = 11); St46–1dps (n = 8); 2dps–3dps (n = 4); *HoxD12Alpha:* St41–43 (n = 7), St44–45 (n = 5); St46–1dps (n = 5); *HoxD12Beta:* St41–43 (n = 4), St44–45 (n = 3); St46–1dps (n = 5), 2dps–3dps (n = 3); *HoxD13Alpha:* St41–43 (n = 6), St44–45 (n = 12); St46–1dps (n = 13), 2dps–3dps (n = 5), 5–15dps (n = 5); *HoxD13Beta:* St41–43 (n = 7), St44–45 (n = 9); St46–1dps (n = 12), 2dps–3dps (n = 7), 5–15dps (n = 4); *HoxD13EF527821:* St41–43 (n = 4), St44–45 (n = 7); St46–1dps (n = 3); *HoxD14Beta:* St41–43 (n = 11), St44–45 (n = 13); St46–1dps (n = 10), 2dps–3dps (n = 3), 5–15dps (n = 3); *Evx2:* St41–43 (n = 10), St44–45 (n = 7); St46–1dps (n = 7), 2dps–3dps (n = 5), 5–15dps (n = 3); *LNP:* St41–43 (n = 6), St44–45 (n = 4); St46–1dps (n = 3); *And1:* St41–43 (n = 4), St44–45 (n = 5); *And1* plus *Sox9:* St41–43 (n = 7), St44–45 (n = 10); *And1* plus *HoxD4:* St44–45 (n = 7); *And1* plus *HoxD8Beta:* St44–45 (n = 7); *And1* plus *HoxD11Beta:* St44–45 (n = 6); *And1* plus *HoxD12Beta:* St44–45 (n = 9); *And1 plus HoxD13Beta:* St44–45 (n = 8). For catshark the total number of *in situ* specimens examined were as follows: *HoxD12:* St28–31 (n = 3); *And1:* St28–31 (n = 3). Sense probe was used with stage-matched specimens as a negative control for antisense hybridization experiments (see Supplemental Fig. 4). Whole mount imaging was done using a Zeiss Discovery.V12 Stereo microscope equipped with an AxioCam MRc5 camera and Zen 2012 (blue edition) software.

### Histology

Embryos used for sectioning were incubated in successive 10%, 20%, and 30% sucrose in PBS solutions for a minimum of 3 hours each at room temperature, and then transferred to a 1:1 solution of TBS Tissue Freezing Medium (Fisher): 30% Sucrose in PBS for 8 hours. Embryos were then embedded in TBS Tissue Freezing Medium in disposable plastic moulds and frozen for 10 minutes using isopentane chilled with liquid nitrogen. Embryos were cryosectioned at 16 μm on a Leica CM1850 cryostat, dried for two hours at 35 °C on a slide warmer, and cover-slipped with 25% glycerol in PBS. Sections were imaged using a Zeiss AxioImager.M2 compound microscope equipped with an Axiocam 503 color camera and Zen 2012 (blue edition) software.

## Additional Information

**How to cite this article**: Tulenko, F. J. *et al*. *HoxD* expression in the fin-fold compartment of basal gnathostomes and implications for paired appendage evolution. *Sci. Rep.* **6**, 22720; doi: 10.1038/srep22720 (2016).

## Supplementary Material

Supplementary Information
