# Supplementary material for: HoxD expression in the fin-fold compartment of basal gnathostomes and implications for paired appendage evolution
Source: Sci Rep. 2016 Mar 4;6:22720. doi: 10.1038/srep22720 (PMC4778128; doi:10.1038/srep22720)
Supplement: Supplementary Information [file srep22720-s1.pdf]

HoxD expression in the fin-fold compartment of basal gnathostomes and implications for paired appendage evolution

Tulenko, Augustus, Massey, Sims, Mazan, and Davis

Supplemental Materials

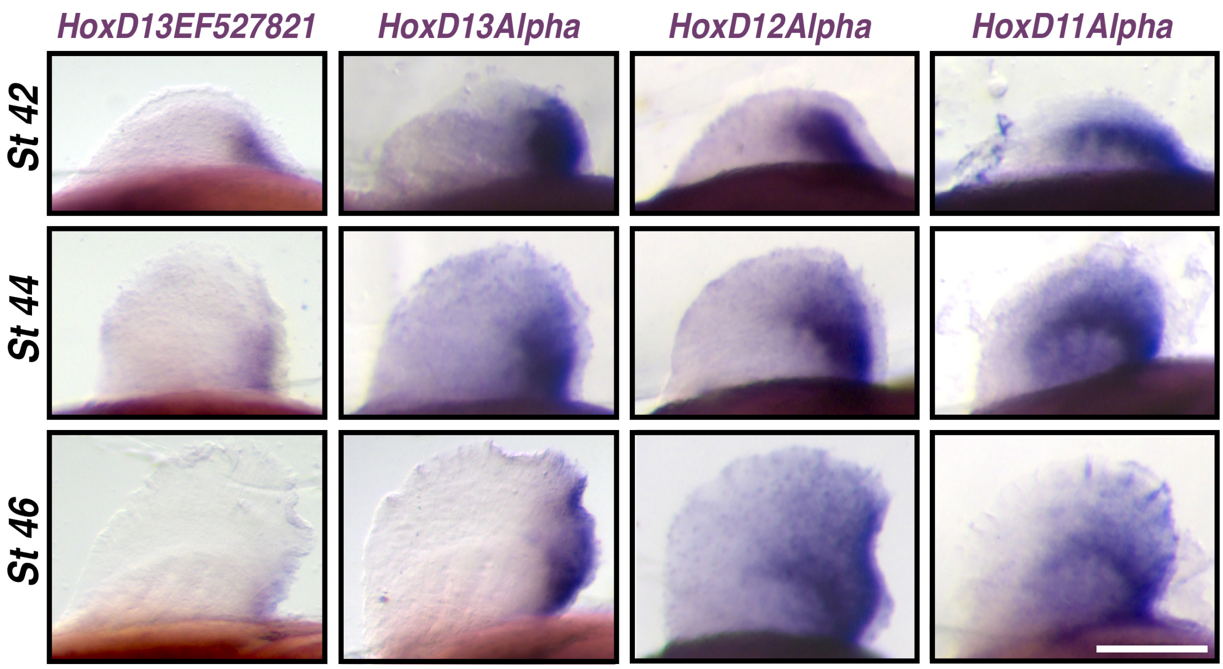

Supplementary Figure 1 | 5' *HoxD* expression for *HoxD13EF527821(Beta)* and *Alpha* paralogues. Pectoral fin whole-mount *in situ* hybridizations for *HoxD13EF527821, HoxD13Alpha*, *HoxD12Alpha*, and *HoxD11Alpha*, from stages 42 (early fin bud) to 46 (differentiated fin – onset of feeding larva). *HoxD13EF527821* is a 3'UTR probe designed against *HoxD13* (Genbank # HoxD13EF527821) as described in reference 22. Compare to expression of *HoxD* cluster paralogues in Fig. 2b. Pectoral fins in ventral view, anterior to the left, distal is up. Scale bars = 200nm

As described in the Introduction and Results, our *in situ* data for coding probes targeted against *Polyodon HoxD13Alpha* and *HoxD13Beta* reveal a posteriorly restricted expression domain at all stages of development examined (Figure 2; Supplementary Figure 1; Supplementary Figure 2). Notably, these results differ from those of a previous study[22], which reported anterior *HoxD13(Beta)* expression along the site of distal radial formation in Stage 46 fins. Because of this apparent difference, we performed whole mount *in situ* hybridizations using the previously published probe (designated as *HoxD13EF527821*), which unlike our coding probes was primarily targeted against the 3'UTR. Significantly, all three *HoxD13* probes assayed showed posteriorly restricted labeling with no evidence of anterior expansion at later stages.

HoxD expression in the fin-fold compartment of basal gnathostomes and implications for paired appendage evolution
Tulenko, Augustus, Massey, Sims, Mazan, and Davis

Supplemental Materials

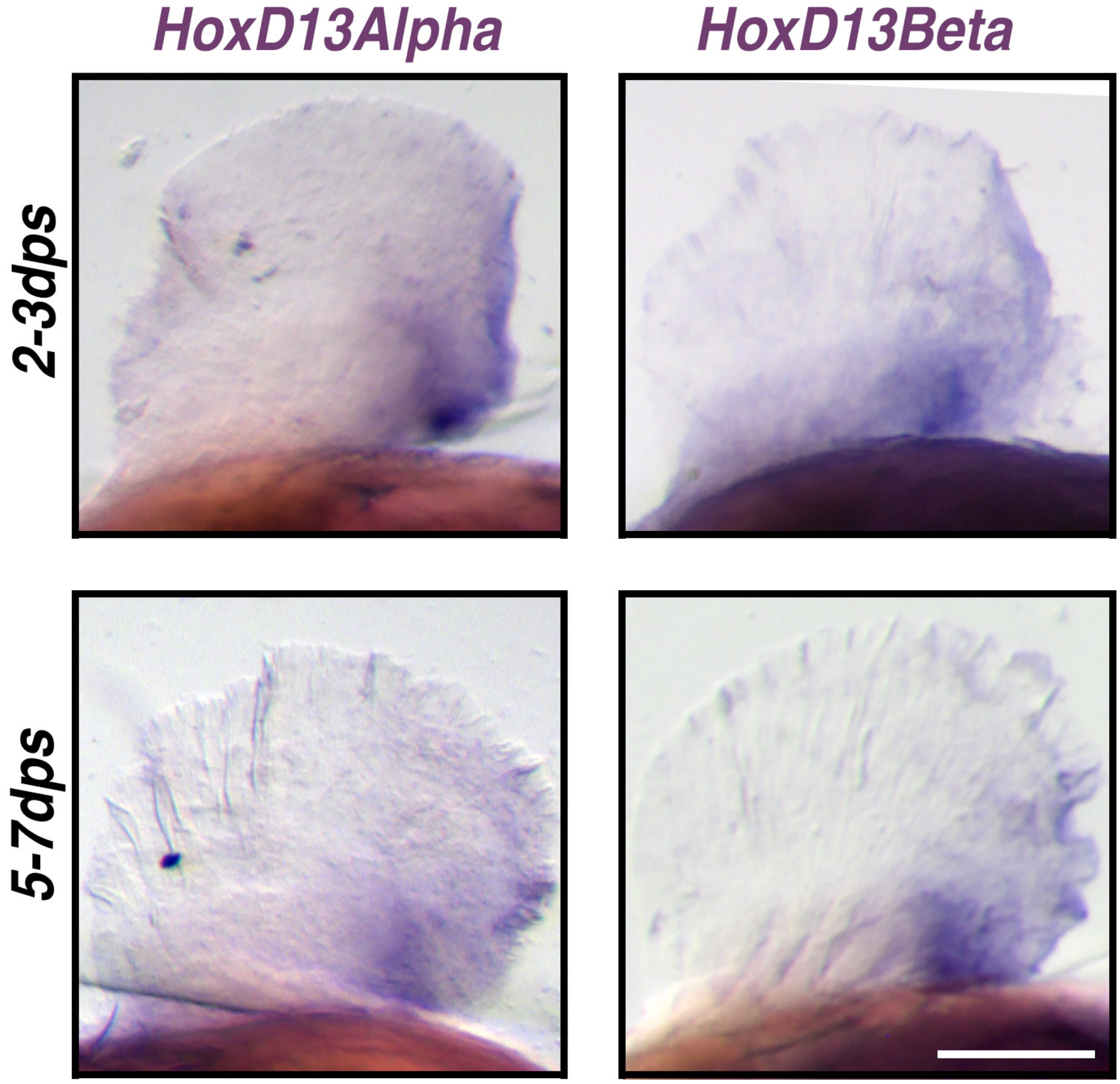

Supplementary Figure 2 | Comparison of *HoxD13* Alpha and Beta paralogues at late stages of development. Pectoral fin whole-mount *in situ* hybridizations for *HoxD13Alpha* and *HoxD13Beta* for two later stages of development: 2-3 days post-staging (dps) and 5-7 dps. The collinear pattern of *HoxD* nesting established in early fin buds was maintained through later stages and no further extension of *HoxD13* expression domains was observed. Pectoral fins in ventral view, anterior to the left, distal is up. Scale bars = 200nm

HoxD expression in the fin-fold compartment of basal gnathostomes and implications for paired appendage evolution

Tulenko, Augustus, Massey, Sims, Mazan, and Davis

Supplemental Materials

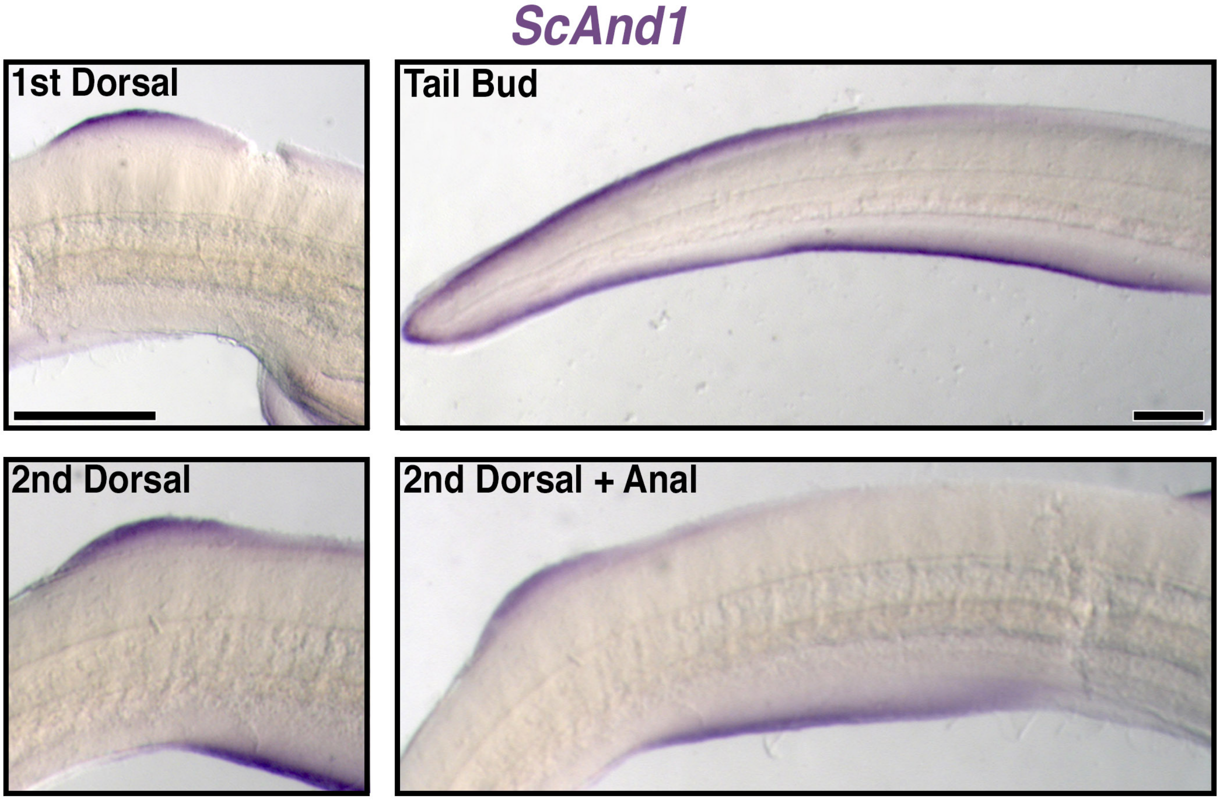

Supplementary Figure 3 | *Actinodin1* homologue (*ScAnd1*) expression in chondrichthyan median fins. Whole-mount *in situ* hybridizations for the catshark *Scyliorhinus canicula Actinodin1* homologue (*ScAnd1*), shown to be an early molecular marker for cells contributing to the fin-fold in teleosts. *ScAnd1* expression in Stage 28 first and second dorsal fins, anal fin, and tail bud. Anterior to right, dorsal is up for all figures. Scale bars = 200nm

HoxD expression in the fin-fold compartment of basal gnathostomes and implications for paired appendage evolution

Tulenko, Augustus, Massey, Sims, Mazan, and Davis

Supplemental Materials

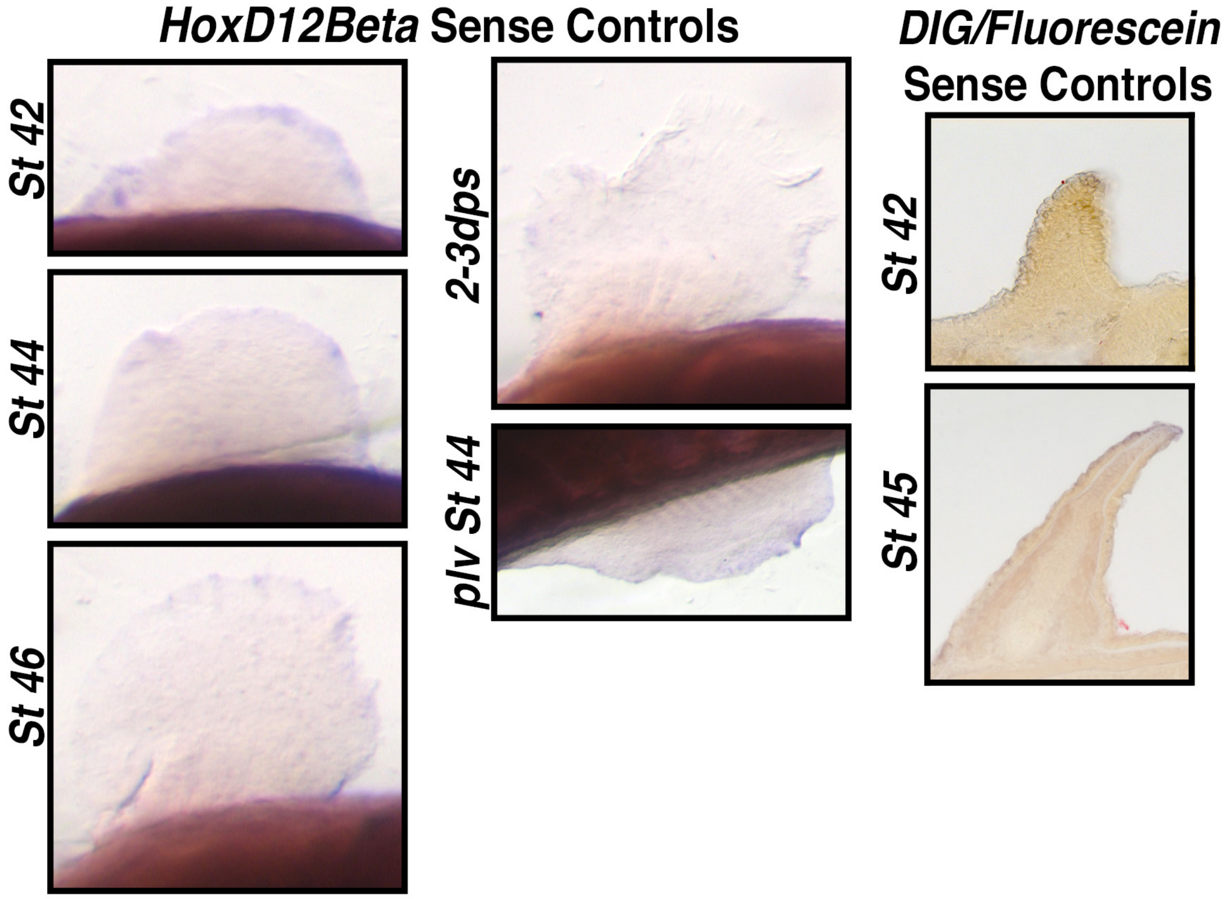

Supplementary Figure 4 | Sense controls for whole mount and sectioned fins. Whole mount *in situ* hybridizations for Sense *HoxD12* (pectoral fins for stages 42-46, 2-3dps, pelvic fin for stage 44), and sections for double colorimetric Sense DIG / Sense Fluorescein labeled pectoral fins (stages 42, 45). Compare whole mounts to Figure 2 and Supplementary Figure 1; and sections to Figure 3b and Figure 4. For whole mounts, pectoral fins in ventral view, pelvic fin in medial view, anterior to the left.
